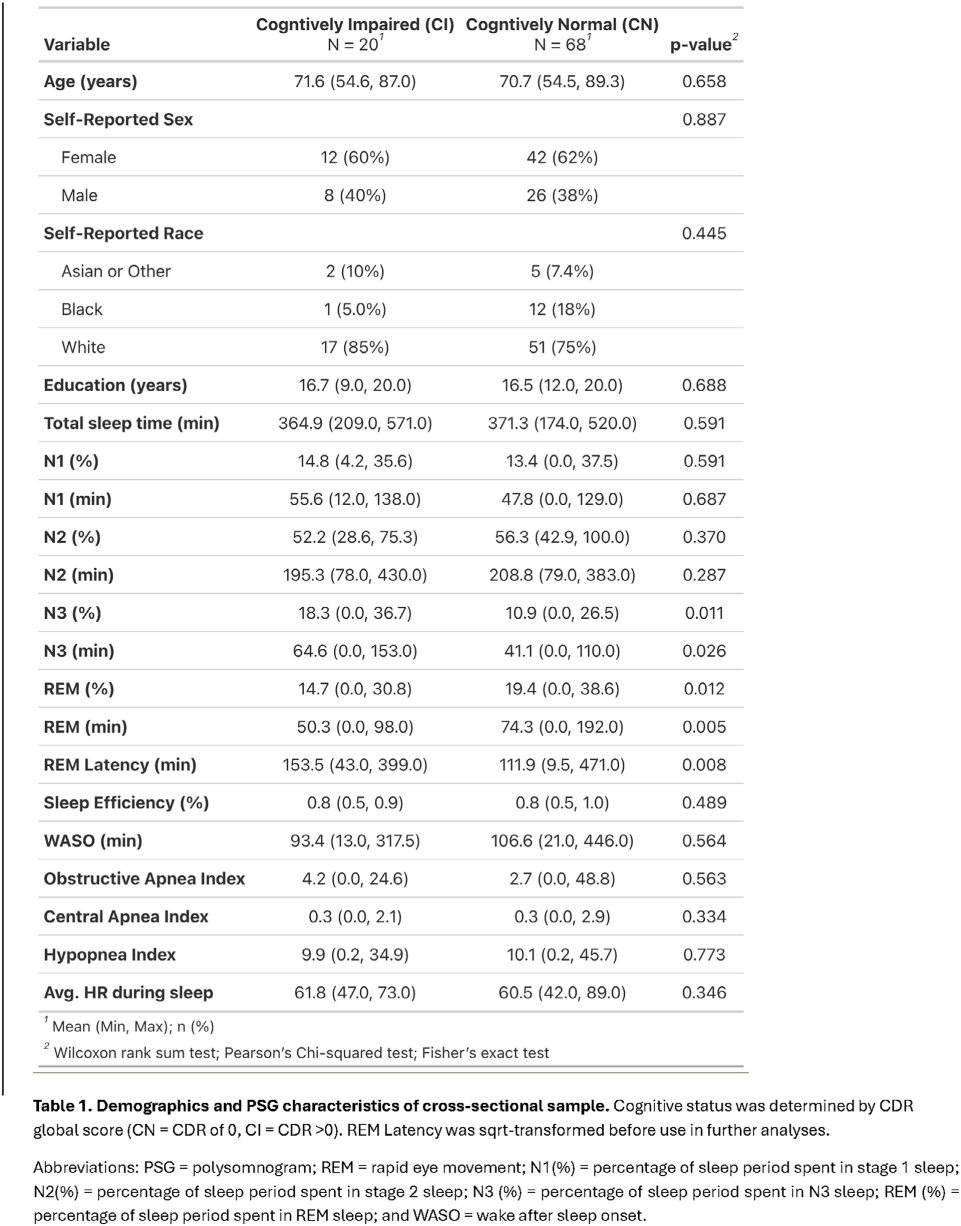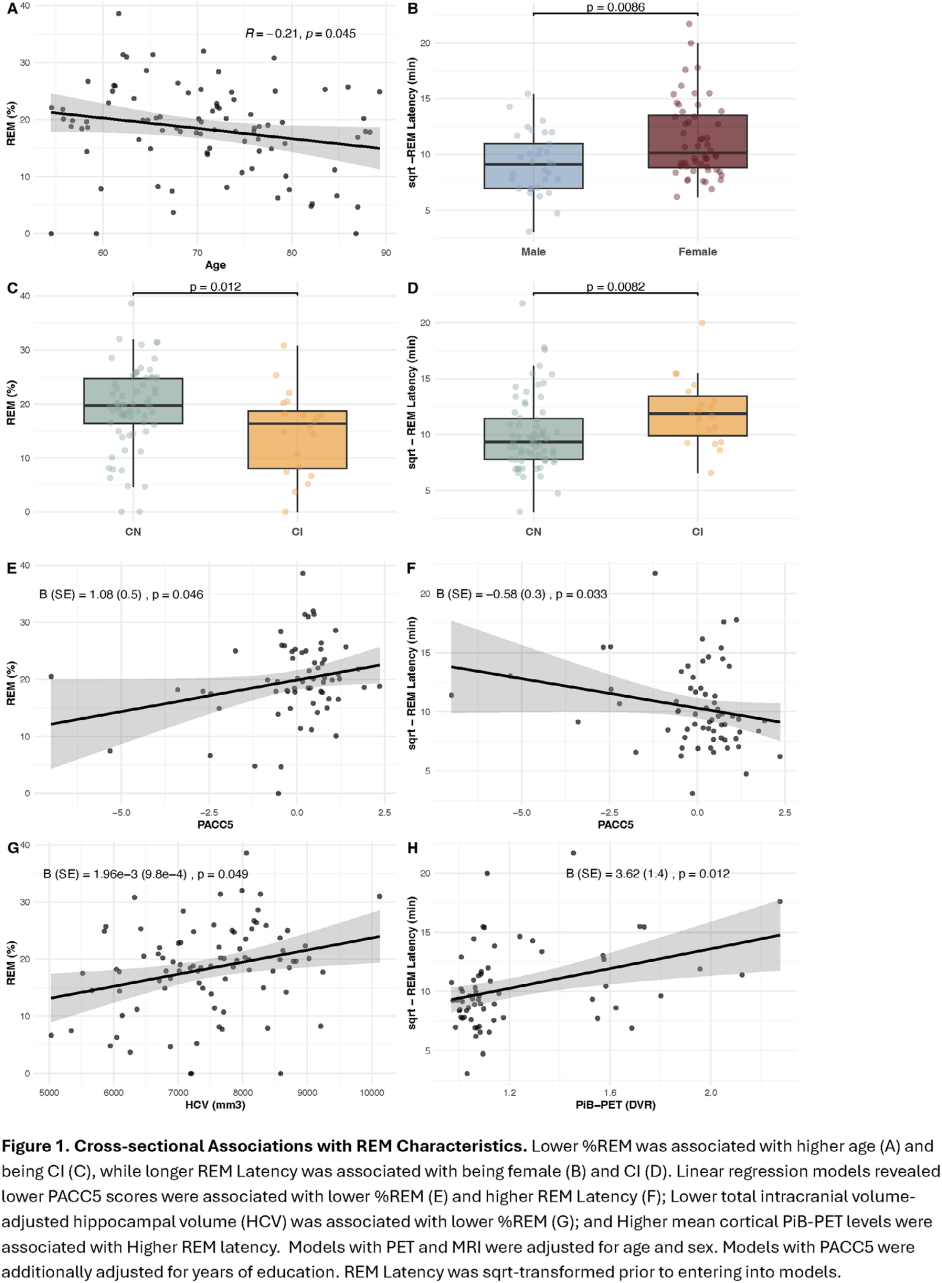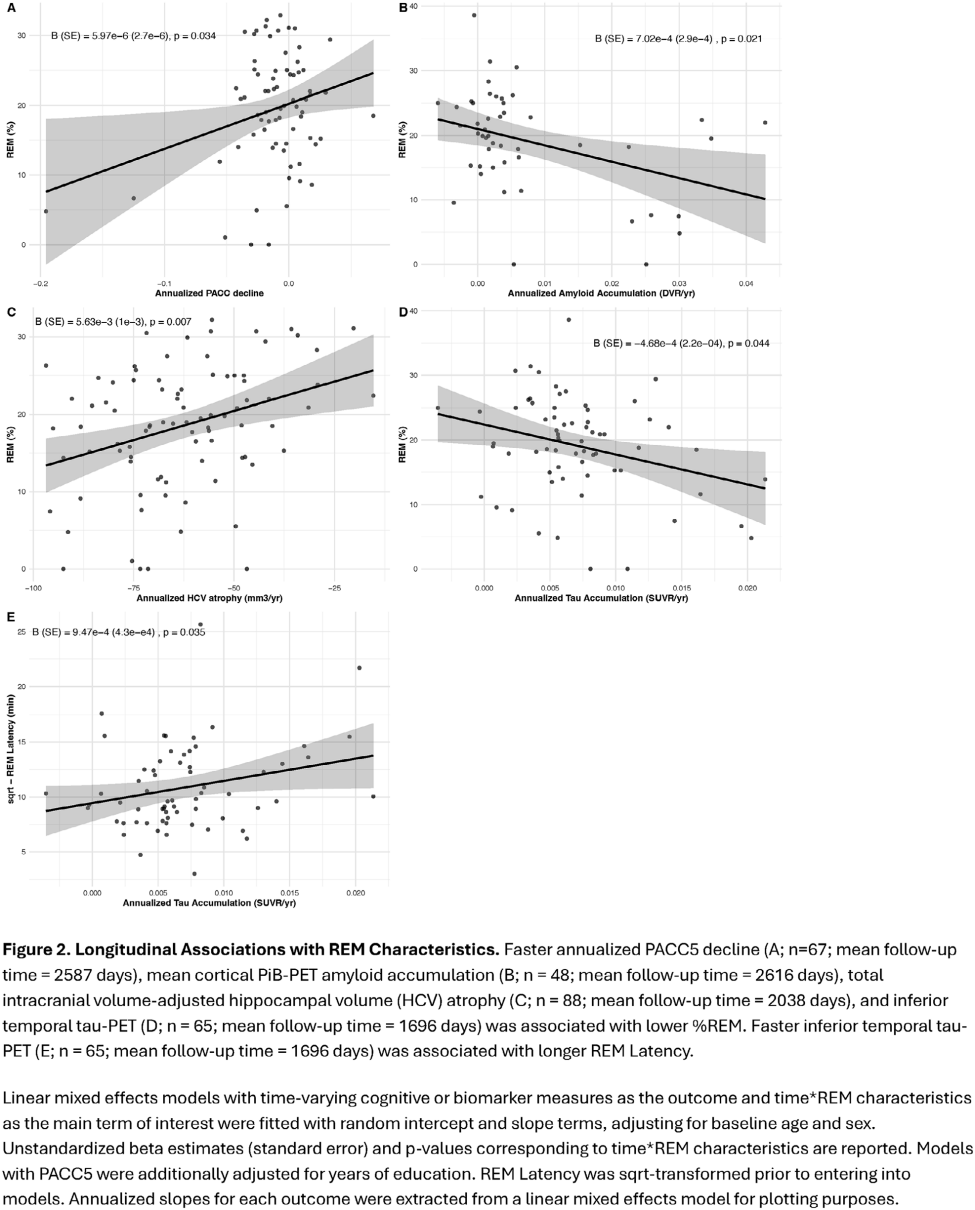# REM Sleep is Associated with Cognition and AD Biomarkers Longitudinally in Cognitively Normal and Impaired Older Adults

**DOI:** 10.1002/alz70861_108080

**Published:** 2025-12-23

**Authors:** Valentina Pinilla, Kailee A. Palmgren, Yiwen Rao, Yazmeen Usman, Michael J. Properzi, Gretchen Reynolds, Wai‐Ying Wendy Yau, Ina Djonlagic, Susan Redline, Shaun Purcell, Keith A. Johnson, Reisa A. Sperling, Jasmeer P. Chhatwal, Stephanie A. Schultz

**Affiliations:** ^1^ Massachusetts General Hospital, Boston, MA USA; ^2^ Department of Neurology, Brigham and Women's Hospital, Boston, MA USA; ^3^ Mass General Brigham, Boston, MA USA; ^4^ Massachusetts General Hospital, Harvard Medical School, Boston, MA USA; ^5^ Beth Israel Deaconess Hospitals, Harvard Medical School, Boston, MA USA; ^6^ Brigham and Women’s Hospital, Harvard Medical School, Boston, MA USA; ^7^ Brigham and Women's Hospital, Harvard Medical School, Boston, MA USA

## Abstract

**Background:**

Sleep disturbances are common among older adults and represent a potentially modifiable risk factor for the development of Alzheimer disease (AD). Previous work from our group and others suggests higher neocortical tau is associated with non‐rapid eye movement sleep characteristics, independent of amyloid levels in cognitively normal (CN) individuals. However, the extent to which changes in rapid eye movement (REM) sleep, a sleep stage critical for memory consolidation, may be related to the accumulation of AD pathology, brain atrophy, and cognitive decline is not well understood.

**Method:**

Eighty‐eight (68 CN and 20 cognitively impaired [CI]) individuals (Table 1) underwent at‐home polysomnography (PSG), as well as cognitive assessment and neuroimaging (mean time from PSG <60 days). Linear regression and linear mixed effects models were used to examine the associations between PSG outcomes of interest (percentage of sleep period spent in REM [%REM] and REM latency [REML]) and cross‐sectional and longitudinal pre‐clinical Alzheimer’s cognitive composite‐5 (PACC5) scores, tau‐positron emission tomography (PET), amyloid‐PET and hippocampal volume (HCV), after adjusting for covariates.

**Result:**

Cross‐sectionally, %REM was lower in CI individuals and with increasing age, and longer REML was associated with being CI and female (Figure 1A‐D). Lower %REM was associated with lower PACC5 scores and lower HCV, and longer REML was associated with lower PACC5 scores and higher amyloid‐PET (Figure 1E‐H). Longitudinally, lower %REM and longer REML were significantly associated with greater decline in PACC5 scores, and HCV, as well as increases in amyloid and tau‐PET (Figure 2).

**Conclusion:**

Overall, REM characteristics differed as a function of age, sex, and clinical status. Across CN and CI individuals, REM characteristics were associated with cognitive performance and AD biomarker status. Longitudinal data demonstrated the rate of change in cognition and AD biomarkers in the time preceding PSG assessment was also strongly associated with REM characteristics. These findings indicate that accumulation of AD pathology over time may negatively impact REM patterns, highlighting the need to better understand the longitudinal relationship between sleep characteristics, AD progression, and cognitive decline, particularly in the case of interventions aimed at improving REM sleep quality and how AD (anti‐amyloid and anti‐tau) therapies impact REM patterns.